# Knowledge Mapping and Global Trends in the Field of the Objective Structured Clinical Examination: Bibliometric and Visual Analysis (2004-2023)

**DOI:** 10.2196/57772

**Published:** 2024-09-30

**Authors:** Hongjun Ba, Lili Zhang, Xiufang He, Shujuan Li

**Affiliations:** 1 Department of Pediatric Cardiology First Afﬁliated Hospital of Sun Yat-sen University Guangzhou China

**Keywords:** Objective Structured Clinical Examination, OSCE, medical education assessment, bibliometric analysis, academic collaboration, health care professional training, medical education, medical knowledge, medical training, medical student

## Abstract

**Background:**

The Objective Structured Clinical Examination (OSCE) is a pivotal tool for assessing health care professionals and plays an integral role in medical education.

**Objective:**

This study aims to map the bibliometric landscape of OSCE research, highlighting trends and key influencers.

**Methods:**

A comprehensive literature search was conducted for materials related to OSCE from January 2004 to December 2023, using the Web of Science Core Collection database. Bibliometric analysis and visualization were performed with VOSviewer and CiteSpace software tools.

**Results:**

Our analysis indicates a consistent increase in OSCE-related publications over the study period, with a notable surge after 2019, culminating in a peak of activity in 2021. The United States emerged as a significant contributor, responsible for 30.86% (1626/5268) of total publications and amassing 44,051 citations. Coauthorship network analysis highlighted robust collaborations, particularly between the United States and the United Kingdom. Leading journals in this domain—*BMC Medical Education*, *Medical Education*, *Academic Medicine*, and *Medical Teacher*—featured the highest volume of papers, while *The Lancet* garnered substantial citations, reflecting its high impact factor (to be verified for accuracy). Prominent authors in the field include Sondra Zabar, Debra Pugh, Timothy J Wood, and Susan Humphrey-Murto, with Ronaldo M Harden, Brian D Hodges, and George E Miller being the most cited. The analysis of key research terms revealed a focus on “education,” “performance,” “competence,” and “skills,” indicating these are central themes in OSCE research.

**Conclusions:**

The study underscores a dynamic expansion in OSCE research and international collaboration, spotlighting influential countries, institutions, authors, and journals. These elements are instrumental in steering the evolution of medical education assessment practices and suggest a trajectory for future research endeavors. Future work should consider the implications of these findings for medical education and the potential areas for further investigation, particularly in underrepresented regions or emerging competencies in health care training.

## Introduction

Objective Structured Clinical Examinations (OSCEs) have emerged as indispensable tools for assessing health care professionals, providing structured evaluations of clinical competencies, communication skills, and decision-making abilities [1,2]. Despite their widespread adoption since the 1970s, the landscape of OSCE research remains multifaceted and dynamic, reflecting ongoing innovations in medical, nursing, and allied health education [3].

While numerous studies have explored various aspects of OSCEs, gaps persist in our understanding of the overarching trends and global dynamics shaping this field. A comprehensive review of the existing literature highlights the need for a systematic approach to mapping the knowledge landscape and identifying emerging trends through bibliometric analysis [4-6]. By applying quantitative methods to scholarly publications, bibliometric analysis offers a unique opportunity to uncover hidden patterns, elucidate research trajectories, and forecast future directions in OSCE research.

Building on this rationale, our study aims to bridge these gaps by conducting a bibliometric analysis of OSCE literature from 2004 to 2023. We hypothesize that this analysis will reveal distinct patterns of publication output, collaboration networks, and thematic clusters within the OSCE research domain. Specifically, we seek to (1) identify key research themes, including but not limited to assessment methodologies, educational interventions, and technological innovations in OSCEs; (2) map the global distribution of OSCE research, highlighting geographic hotspots and areas of collaboration; and (3) explore the interconnections between different disciplines within medical education, shedding light on interdisciplinary collaborations and knowledge diffusion.

By elucidating these aspects, our study aims to provide stakeholders in medical education with valuable insights into the current state and future directions of OSCE research. Ultimately, this knowledge mapping exercise seeks to inform evidence-based decision-making, guide educational practices, and stimulate further research in the field of clinical skills assessment.

## Methods

### Data Acquisition and Search Strategy

The bibliographic accuracy of literature types in the Web of Science Core Collection (WoSCC) database is superior to any other database, making it the optimal choice for conducting literature analysis [7,8]. Therefore, we opted to perform our search within this database. We conducted a search in the Web of Science (WoS) for all relevant papers published between January 1, 2004, and December 31, 2023. The search formula “(TS=(The Objective Structured Clinical Examination)) or TS=(OSCE)” was used. The literature screening for this study was based on the inclusion criteria: (1) full-text publications related to the OSCEs; (2) papers and review manuscripts written in English; and (3) papers published between January 1, 2004, and December 31, 2023. The exclusion criteria included (1) topics not related to the OSCEs and (2) papers in the form of conference abstracts, news briefs, and so on. A plain text version of the papers was exported.

### General Data

**Figure 1:**
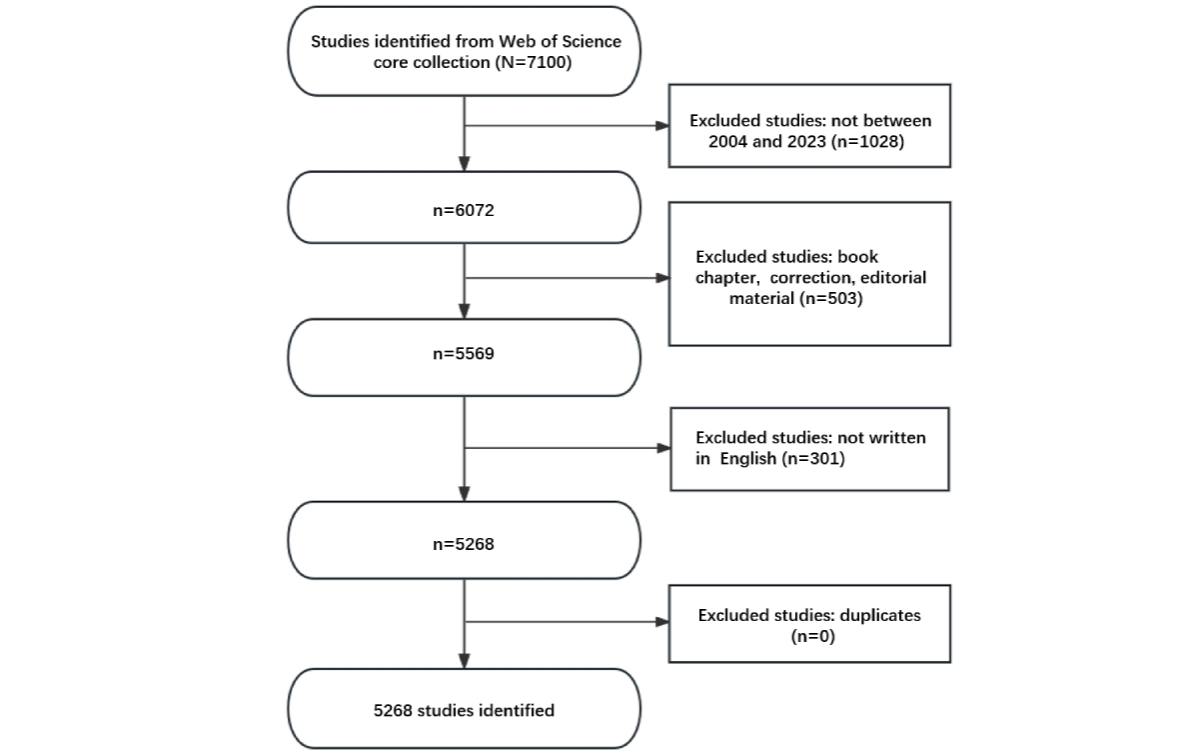
The workflow of data collection and bibliometric analysis.

Figure 1 shows the process of literature searching and bibliometric analysis. The results indicate that from January 1, 2004, to December 31, 2023, there were a total of 5268 publications related to the OSCE in the WoSCC database, including 1800 papers (84.96%) and 384 reviews (15.04%). The literature involved 133 countries and regions, 5291 institutions, and 24,478 authors.

### Data Analysis

To depict annual publication trends and the distribution of national contributions, we used GraphPad Prism (version 8.0.2; Dotmatics). For the bibliometric analysis and the visualization of scientific knowledge maps, the study used both CiteSpace (6.2.4R, 64 bit advanced edition; Chaomei Chen, Drexel University) [9] and VOSviewer (version 1.6.18; Leiden University) [10]. These tools were selected for their robustness in handling extensive bibliometric data and their ability to graphically represent complex networks.

VOSviewer, a Java-based software pioneered by van Eck and Waltman [9] in 2009, facilitates the construction of various types of network maps, such as bibliographic coupling, cocitation, and coauthorship networks. CiteSpace, developed by Professor Chaomei Chen, provides a dynamic and computer-based platform for identifying and visualizing patterns and trends in scientific literature, thereby enabling the exploration of knowledge domains and predictive analysis of research trajectories [10].

Our methodological approach within these applications involved setting specific parameters for network density, threshold values for the inclusion of nodes, and time-slicing techniques to analyze temporal changes. The references corresponding to the software applications were verified against our citation list to ensure accuracy [9,10].

In our study using VOSviewer and CiteSpace software tools for bibliometric analysis, the criteria for defining country-based collaborations were established based on specific considerations. Collaborations were determined by considering the first authors and corresponding authors listed in the paper bylines. This approach was chosen to ensure inclusivity and to capture the entirety of collaborative efforts between researchers from different countries.

The burst detection in CiteSpace is based on the Kleinberg algorithm, which is based on modeling the stream using an infinite-state automaton to extract a meaningful structure from document streams that arrive continuously over time [11]. These analyses can show the fast-growing topics that last for multiple years as well as a single year.

### Rationale for Analysis Selection

The aforementioned techniques were chosen a priori due to their widespread use and effectiveness in bibliometric studies. They provide robust and complementary insights into productivity, impact, and collaborative patterns within the research field.

## Results

### Publication Trend

**Figure 2:**
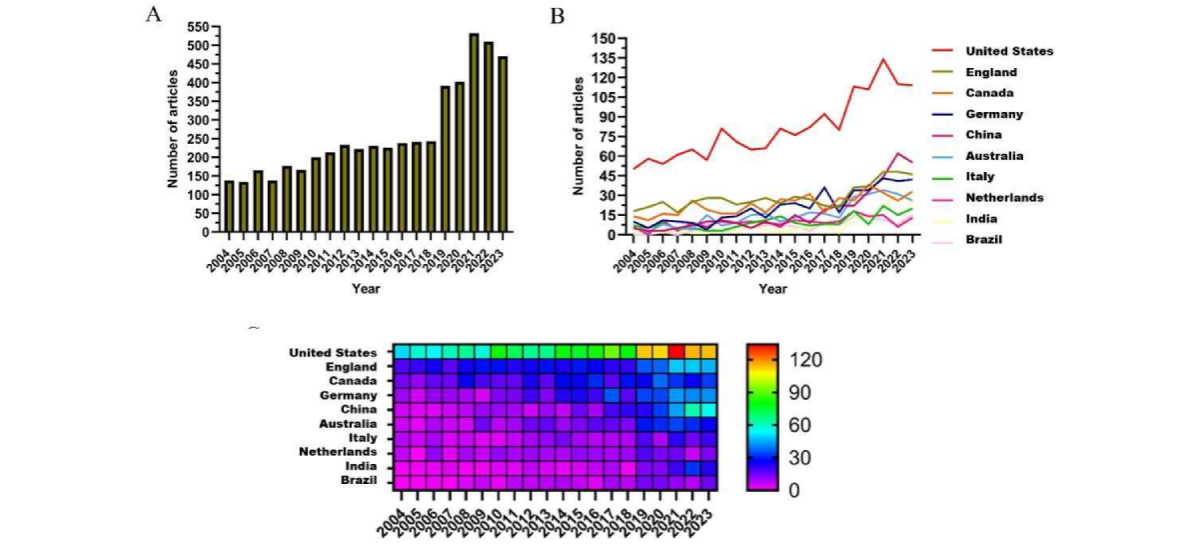
Trend chart of publications in the past 20 years. (A) Annual publication count chart. (B) Line chart of national publication count. (C) Heatmap of national publication count.

Since 2004, there has been a gradual increase in the number of papers published annually (Figure 2A). We have divided this into 3 periods: from 2004 to 2010, there was a slow growth, with fewer than 150 papers published per year, indicating that the field had not yet captured researchers’ attention. From 2011 to 2018, the volume of publications gradually increased, indicating growing interest in the field. After 2019, there was a rapid rise in the number of publications, peaking in 2021, which suggests that the field has received widespread attention since then.

### Country or Region and Institution Contributions

Figure 2B and C show the annual number of publications from the top 10 countries over the past decade. The top 5 countries in the field are the United States, the United Kingdom, Canada, Germany, and China, respectively. The United States accounts for 30.86% (1626/5268) of the total volume of publications, significantly surpassing other countries.

**Figure 3:**
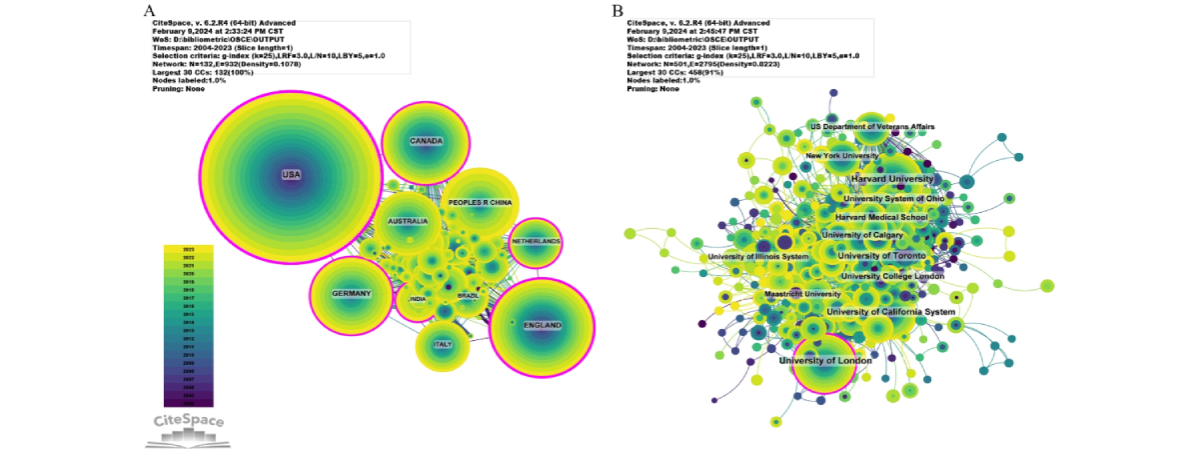
Network graph of national and institutional collaborations. (A) Network graph of national collaborations. (B) Network graph of institutional collaborations. The bubble size represents the number of publications. WoS: Web of Science.

Among the top 10 countries or regions in terms of the number of published papers, the United States had a citation count of 44,051, far exceeding all other countries or regions. Its citation-per-publication ratio (27.13) ranks third among all countries or regions, which suggests a generally high quality of the published papers. The United Kingdom had the second-highest number of published papers (576 papers) and ranked second in terms of citation count (15,929 citations). The cooperation network, as shown in Figure 3A, indicates close collaboration between the United States and the United Kingdom, which are the highest producers.

A total of 5291 institutions have systematically published papers related to the OSCE. Among the top 10 institutions in terms of publication volume, 6 are from the United States, 2 are from the United Kingdom, and 2 are from Canada (Figure 3B).

### Journals’ Contributions

Tables 1 and 2 list the top 10 journals with the highest outputs and the most citations, respectively. *BMC Medical Education*, with 227 out of 5268 papers, accounting for 4.31% of publications in the field, is the journal with the most published papers, followed by *Medical Teacher* (179/5268, 3.40%), *Medical Education* (132/5268, 2.51%), and *Journal of Surgical Education* (66/5268, 1.25%). Among the top 10 most productive journals, *Annals of the Rheumatic Diseases* has the highest impact factor at 27.6. All journals are categorized within either Q1 or Q2 quartiles.

The influence of a journal is determined by the frequency with which it is cocited, which indicates whether the journal has made a significant impact on the scientific community. According to Table 2, the most commonly cocited journal is *Medical Education* with 1868 citations, followed by *Academic Medicine* with 1775 citations, and *Medical Teacher* with 1597 citations. Among the top 10 journals by cocitation count, *The Lancet* was cited 697 times and has the highest impact factor of 168.9 within these top journals. All journals within the most cocited list are in the Q1 or Q2 zone.

**Table 1:** Top 10 most productive journals.

Rank	Journals	Papers (N=5268), n (%)	IF^a^	Quartile in category
1	*BMC Medical Education*	227 (4.31)	3.6	Q1
2	*Medical Teacher*	179 (3.40)	4.7	Q1
3	*Medical Education*	132 (2.51)	7.1	Q1
4	*Journal of Surgical Education*	66 (1.25)	2.9	Q2
5	*Academic Medicine*	64 (1.21)	7.4	Q1
6	*Patient Education and Counseling*	64 (1.21)	3.5	Q2
7	*Advances in Health Sciences Education*	60 (1.14)	4.0	Q1
8	*American Journal of Pharmaceutical Education*	59 (1.12)	3.3	Q2
9	*PLoS One*	59 (1.12)	3.7	Q2
10	*Nurse Education Today*	56 (1.06)	3.9	Q1

^a^IF: impact factor.

**Table 2:** Top 10 journals with the highest number of cocitations. Cocited journals refer to 2 or more journals that are simultaneously cited in the reference lists of other research papers.

Rank	Cited journals	Cocitations, n	IF^a^ (2020)	Quartile in category
1	*Medical Education*	1868	4.7	Q1
2	*Academic Medicine*	1775	7.4	Q1
3	*Medical Teacher*	1597	4.7	Q1
4	*BMC Medical Education*	941	3.6	Q1
5	*JAMA—Journal of American Medical Association*	931	120.7	Q1
6	*British Medical Journal*	827	107.7	Q1
7	*Advances in Health Sciences Education*	802	4.0	Q1
8	*The Lancet*	697	168.9	Q1
9	*New England Journal of Medicine*	694	158.5	Q1
10	*Teaching and Learning Medicine*	599	2.5	Q3

^a^IF: impact factor.

### Authors and Cocited Authors' Contributions

**Figure 4:**
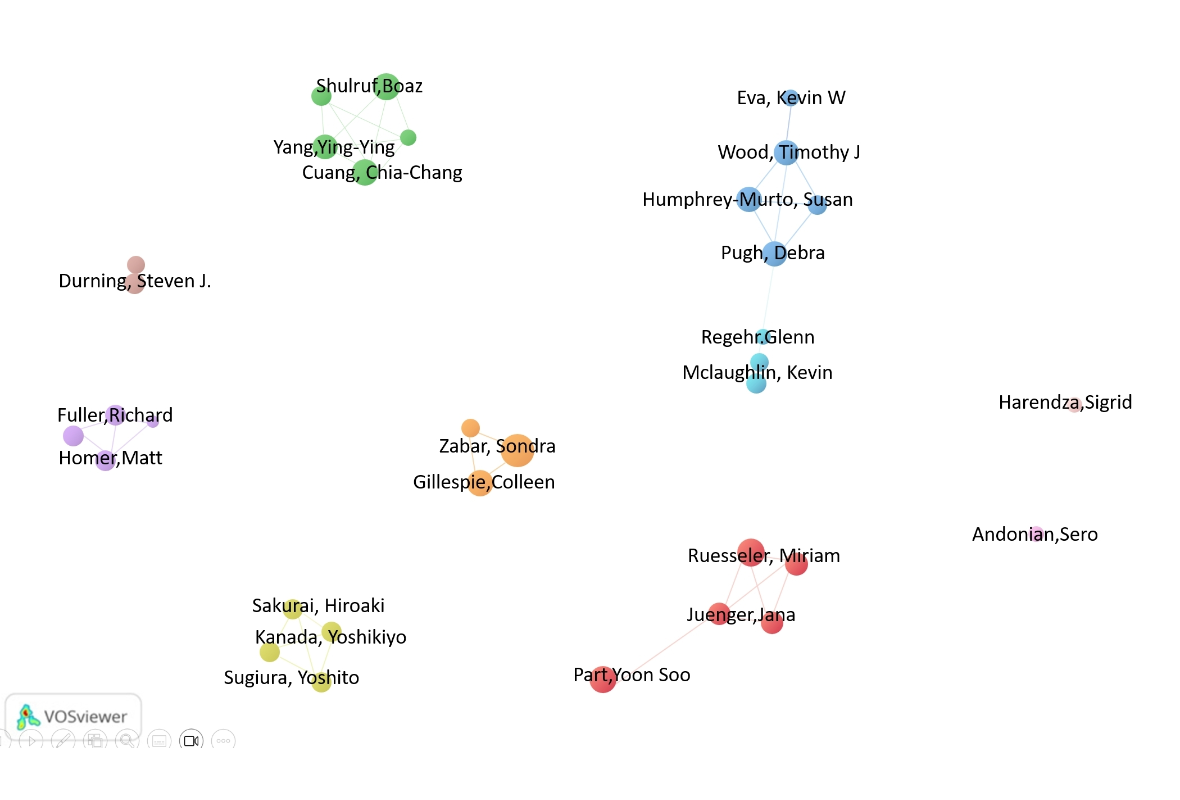
Network diagram of author collaborations. The bubble size represents the number of publications.

Among all authors who have published literature related to OSCE, Tables 3 and 4 list the top 10 authors with the most published papers. Together, these top 10 authors have published 185 papers, accounting for 3.51% of all papers (N=5268) in the field. Sondra Zabar has 26 publications, which is the highest number of published research papers, followed by Debra Pugh with 22, Timothy J Wood with 20, and Susan Humphrey-Murto with 19. Further analysis indicates that among the top 10 ranked authors, 4 are from the United States, 3 are from Canada, 2 are from Australia, and 1 is from China. CiteSpace visualizes the network of relationships between authors (Figure 4).

Table 4 displays the top 10 authors who have been cocited and cited the most, respectively. A total of 148 authors have been cited more than 50 times, indicating that their research has a high reputation and influence. The largest nodes are associated with the authors who have been cocited the most, including Ronald M Harden with 751 citations, Brian D Hodges with 330 citations, and George E Miller with 222 citations.

**Table 3:** Top 10 most productive authors.

Rank	Authors	Papers, n	Locations
1	Zabar, Sondra	26	United States
2	Pugh, Debra	22	Canada
3	Wood, Timothy J	20	Canada
4	Humphrey-Murto, Susan	19	Canada
5	Gillespie, Colleen	17	United States
6	Shulruf, Boaz	17	Australia
7	Yang, Ying-Ying	17	China
8	Durning, Steven J	16	United States
9	Fuller, Richard	16	Australia
10	Park, Yoon Soo	15	United States

**Table 4:** Top 10 most cocited authors.

Rank	Cocited authors	Citations, n
1	Harden, Ronald M	751
2	Hodges, Brian D	330
3	Miller, George E	222
4	Epstein, Ronald M	194
5	van der Vleuten, Cees PM	173
6	Wass, Valerie	172
7	Khan, Kamran Z	164
8	Regehr, Glenn	162
9	Cook, David A	160
10	Downing, Steven M	156

### Analysis of Highly Cited References

**Figure 5:**
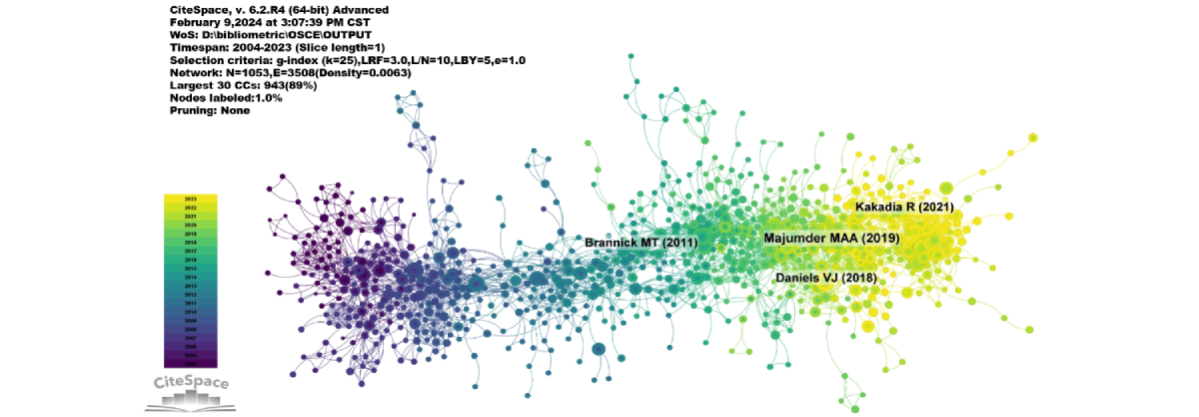
Network diagram of cocited references.

Over the time span from 2004 to 2023, the cocitation network comprised 1053 nodes and 3508 links (Figure 5). According to the top 10 papers by cocitation frequency (Table 5), the most cocited reference is from the journal *Advances in Medical Education and Practice* (impact factor=2.0), titled “An evaluative study of Objective Structured Clinical Examination (OSCE): students and examiners perspectives” [12]. The first author of this paper is Md Anwarul Azim Majumder. The paper posits that OSCE is the gold standard and universal form for assessing medical students’ clinical competence in a comprehensive, reliable, and effective manner.

**Table 5:** Top 10 highest cited references.

Rank	Titles	Journals	IF^a^ (2021)	First authors	Total citations, n
1	An evaluative study of Objective Structured Clinical Examination (OSCE): students and examiners perspectives [12]	*Advances in Medical Education and Practice*	2.0	Majumder, Md Anwarul Azim	38
2	Implementing an online OSCE during the COVID-19 pandemic [13]	*Journal of Dental Education*	2.3	Kakadia, Rahen	31
3	Diagnostic and statistical manual of mental disorders [14]	*Psychiatry Research*	11.3	Mittal, Vijay A	31
4	A systematic review of the reliability of Objective Structured Clinical Examination scores [15]	*Medical Education*	7.1	Brannick, Michael T	30
5	Twelve tips for developing an OSCE that measures what you want [16]	*Medical Teacher*	4.7	Daniels, Vijay John	30
6	Is the OSCE a feasible tool to assess competencies in undergraduate medical education? [17]	*Medical Teacher*	4.7	Patricio, Madalena F	29
7	Techniques for measuring clinical competence: Objective Structured Clinical Examinations [18]	*Medical Education*	7.1	Newble, David	26
8	Assessment in medical education [19]	*New England Journal of Medicine*	158.5	Epstein, Ronald M	26
9	Assessing communication skills of medical students in Objective Structured Clinical Examinations (OSCE)-a systematic review of rating scales [20]	*PLoS One*	3.7	Cömert, Musa	26
10	Twelve tips for conducting a virtual OSCE [21]	*Medical Teacher*	4.7	Hopwood, Jenny	26

^a^IF: impact factor.

### Keyword Analysis

Through the analysis of keywords, we can quickly understand the situation and development direction of a field. Based on the co-occurrence of keywords in VOSviewer, the hottest keyword is “education” (n=677 occurrences), followed by “performance” (n=536), “competence” (n=458), and “skills” (n=449; Table 6).

**Table 6:** Top 20 keywords co-occurrence frequencies.

Rank	Keywords	Co-occurrences, n
1	Education	677
2	Performance	536
3	Competence	458
4	Skills	449
5	Reliability	371
6	Assessment	342
7	Students	337
8	Validity	329
9	Simulation	284
10	Medical education	264
11	Diagnosis	228
12	Care	217
13	Prevalence	207
14	Medical students	197
15	Management	196
16	Medical education	171
17	Curriculum	168
18	Communication	161
19	Impact	156
20	Clinical skills	147

### The Burst of Cocited References and Keywords

**Figure 6:**
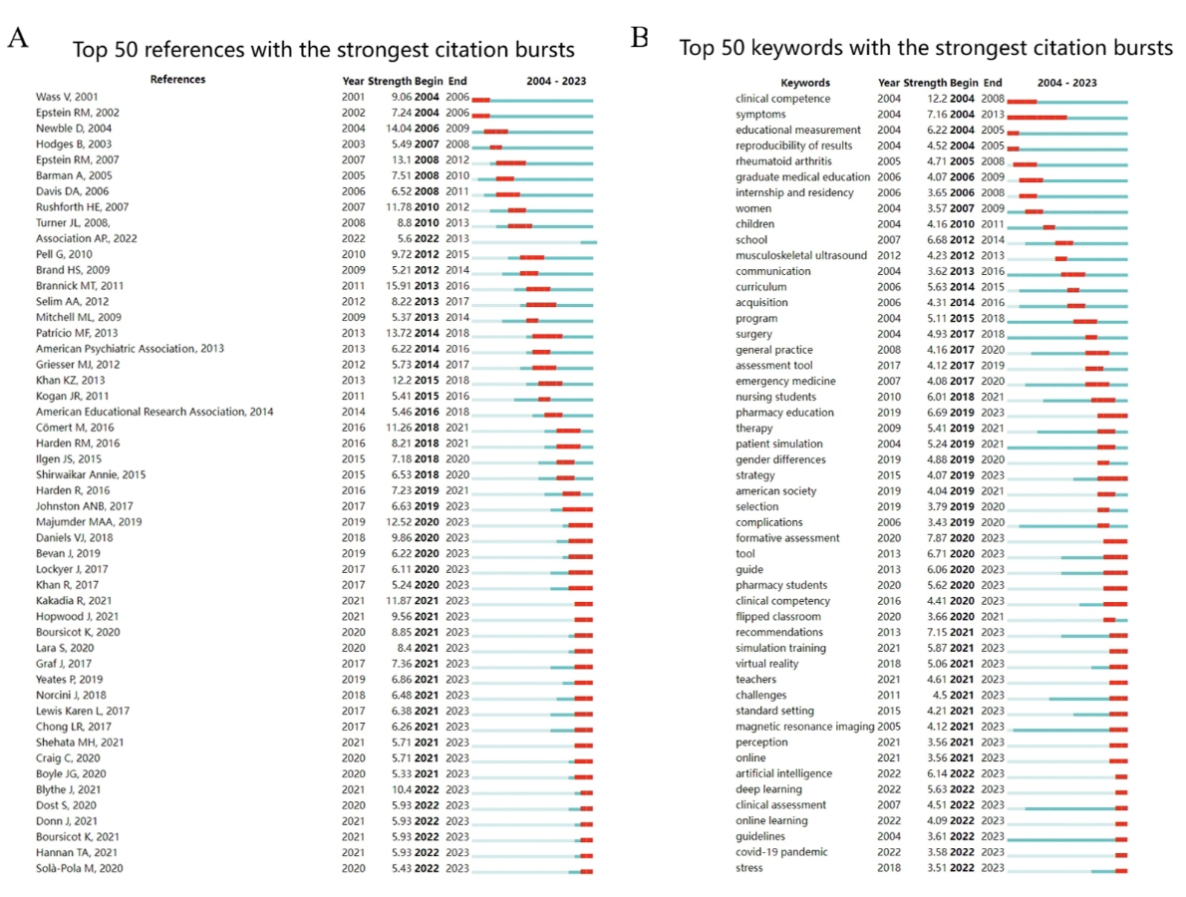
Citation burst graph (A), and keyword burst graph (B; sorted by the beginning year of the burst). The blue bars mean the reference has been published; the red bars mean citation burstness.

With CiteSpace, we identified 50 of the most reliable citation bursts in the field related to OSCE [12,13,15-62]. The most frequently cited reference, with a burst strength of 15.91, is a paper published in *Medical Education* titled “A systematic review of the reliability of Objective Structured Clinical Examination scores” [15], whose first author is Michael T Brannick. The paper suggests that OSCEs consist of a series of simulated tasks to assess medical practitioners’ skills in diagnosing and treating patients. Of the 50 references, 47 (94%) were published between 2004 and 2023, indicating that these papers have been frequently cited over nearly 20 years. Notably, 24 of these papers are currently at a citation peak (Figure 6A [12,13,15-62]), meaning that research related to OSCE is expected to continue receiving significant attention in the future.

Among the 768 strongest emerging keywords in the field, we focused on the 50 with the most significant surges (Figure 6B), representing the current hotspots in the field and likely future research directions.

## Discussion

### Principal Findings

This study is pioneering in its bibliometric approach to OSCE, encapsulating a comprehensive view of the dynamic research trends in this field. By analyzing the bibliometric data internationally, we have mapped out collaboration networks, identified prevailing research directions, and forecasted potential future developments in OSCE scholarship. The surge in OSCE-related publications since 2019 underscores the recognition of OSCEs as essential for evaluating health care practitioners, meeting the demands of modern medicine for more robust and comprehensive assessment methods to gauge clinical competency [22,63].

Despite this growth, the concentration of research output in countries like the United States, the United Kingdom, and Canada may reflect deeper issues of resource allocation and priority setting in medical education globally [64,65]. This suggests a need for a more nuanced discussion on the uneven geographical spread of OSCE research and its implications. The disparity in research contribution could hinder the global exchange of innovative practices and perspectives in medical education [66,67].

Furthermore, the bibliometric data point to the importance of technology in OSCEs, particularly the integration of virtual and augmented reality. However, to fully understand the implications of technological advances, a more detailed analysis is warranted. This should include how technology shapes the development of OSCEs, its impact on the validity and reliability of assessments, and the potential barriers to its widespread adoption [68-70].

The high concentration of publications in Q1 and Q2 quartile journals, especially those with a significant impact factor, attests to the intersection of OSCE research with impactful clinical education and outcomes. The association with prestigious journals underlines the extensive influence and critical importance of OSCEs across multiple medical specialties [71-73].

The prominence of a core group of scholars leading OSCE research suggests a centralization of expertise that could be diversified through broader international collaboration. Such collaboration could introduce various cultural and pedagogical perspectives into the OSCE discourse, thereby enriching both the practice and the research of OSCEs worldwide [74,75].

The keyword analysis reflects a continual focus on the foundational elements of clinical education, such as “education,” “performance,” “competence,” and “skills,” which are at the heart of the OSCE methodology. Emerging research trends suggest a shift toward the integration of innovative educational technologies and methodologies, enhancing both the OSCE process and its outcomes [76,77].

### Comparison to the Literature

Our findings align with those of Lim et al [78], who identified issues with construct, content, and predictive validity in OSCEs in pharmacy education, as well as significant resource challenges. These concerns are echoed in our analysis, where similar validity issues and logistical constraints were observed. Other studies, such as those by Hodges et al [79], have highlighted persistent challenges in psychiatric OSCEs, emphasizing the need for continuous refinement and adaptation. Our study extends these discussions by mapping global trends and collaboration networks, underscoring the necessity for continuous re-evaluation and innovation in OSCE methodologies.

### Implications of Findings

The challenges associated with OSCEs suggest a need for evolving assessment methods that incorporate simulations, peer assessments, and reflective practices. The resource-intensive nature of OSCEs underscores the necessity for scalable and sustainable alternatives, such as virtual simulations. Policymakers and educators should leverage global collaboration networks to share best practices and develop adaptable, technology-enhanced assessment frameworks. This approach will help address validity concerns and logistical constraints, ensuring that educational assessments remain robust and relevant in the ever-evolving landscape of health care education.

### Limitations

Our bibliometric analysis has limitations that may affect our findings. We only used data from the WoSCC database, potentially excluding studies not indexed there and leading to bias toward English-language literature. This limits the scope of our analysis and overlooks valuable contributions from non-English sources.

### Suggestions

To address this, future research should involve a wider range of databases and languages [80,81]. Moreover, the data quality in our study may vary, affecting the credibility of our knowledge mapping. Therefore, caution is needed when interpreting results, and complementary research methods should be considered for a more comprehensive understanding of the field. Longitudinal studies are crucial to assess the impact of OSCEs on medical performance, connecting educational assessments with clinical practice and patient care [82,83].

Moreover, understanding how OSCEs adapt to different health care systems, cultural contexts, and specializations will provide insights into their scalability and adaptability. This is particularly relevant as the health care sector grapples with rapid changes and as medical education seeks to prepare health care professionals for diverse practice environments [19,84].

### Conclusions

In conclusion, this bibliometric study not only reaffirms the enduring importance and evolutionary path of OSCEs within medical education but also emphasizes the need for OSCEs to evolve in step with broader health care transformations. The data-driven insights from this analysis should inform future research directions, influence policymaking, and refine educational strategies. By doing so, OSCEs can continue to serve as a dynamic, relevant, and innovative tool in the arsenal of clinical education and evaluation methods.

## Abbreviations

OSCE: Objective Structured Clinical Examination
WoS: Web of Science
WoSCC: Web of Science Core Collection

## Data Availability

All data generated or analyzed during this study are included in this published article.
